# Native cardiac reserve predicts survival in acute post infarction heart failure in mice

**DOI:** 10.1186/1476-7120-5-46

**Published:** 2007-12-02

**Authors:** Margareta Scharin Täng, Truls Råmunddal, Malin Lindbom, Elmir Omerovic

**Affiliations:** 1 Department of Molecular and Clinical Medicine, Institute of Medicine at Sahlgrenska Academy, Göteborg University, Göteborg, Sweden

## Abstract

Cardiac reserve can be used to predict survival and outcome in patients with heart failure. The aim of this study was to investigate if native cardiac reserve could predict survival after myocardial infarction (MI) in mice.

**Method:**

We investigated 27 healthy C57Bl6 mice (♂10–12 weeks old) with echocardiography using a high-frequency 15-MHz linear transducer. Investigations were performed both at rest and after pharmacological stress induced by dobutamine (1 μg/g body weight i.p.). The day after the echocardiography examination, a large MI was induced by ligation of the left anterior descending (LAD) coronary artery for evaluation of mortality rate.

**Results:**

Two weeks after induction of MI, 7 mice were alive (26%). Evaluation of the difference between the surviving and deceased animals showed that the survivors had a better native ability to increase systolic performance (ΔLVESd -1.86 vs -1.28mm p = 0.02) upon dobutamine challenge, resulting in a better cardiac reserve (ΔFS 37 vs 25% p = 0.02 and ΔCO 0.27 vs -0.10 ml/min p = 0.02) and a better chronotropic reserve (ΔR-R interval -68 vs -19 ms p < 0.01). A positive relationship was found between ability to survive and both cardiac (p < 0.05) and chronotropic reserve (p < 0.05) when the mice were divided into three groups: survivors, surviving < 7 days, and surviving < 1 day.

**Conclusion:**

We conclude that before MI induction the surviving animals had a better cardiac function compared with the deceased. This indicates that native cardiac and chronotropic reserve may be an important determinant and predictor of survival in the setting of large MI and post-infarction heart failure.

## Background

Dobutamine stress echocardiography (DSE) is a well-established method to investigate cardiac reserve in humans [1]. Cardiac reserve can be used to predict survival and outcome in patients with cardiomyopathy [2-4] and provides prognostic information to predict all-cause mortality and cardiac events in elderly patients [5]. A positive response during DSE is also associated with a better clinical outcome and prognosis in patients with acute myocardial infarction (MI) treated with coronary angioplasty [6]. Furthermore, a poor chronotropic reserve has been shown to predict mortality in cardiovascular disease [7,8]. DSE has been widely used in several animal species to study ischemia, and it has been shown that mice (WT) can maintain a positive inotropic response to dobutamine 8 weeks after MI [9]. Evaluation of cardiac reserve during stress provides more information about function and capacity than can be obtained merely by investigations during rest.

The possibility to use cardiac reserve as a prognostic tool before MI has not been studied. The aim of this study was therefore to investigate if native cardiac reserve, assessed by DSE, could predict survival after MI in mice.

## Methods

### Animals

27 healthy male C57BL6 mice, 10–12 weeks of age and with a body weight of 29 ± 3 g underwent DSE. The animals were housed under constant temperature (21–25°C) and in a 12 h light/dark cycle and maintained on water and food *ad libitum*. This experiment has been carried out with approval of the regional Animal Ethics Committee at Göteborg University, Göteborg, Sweden.

### Anesthesia

**Figure 1:**
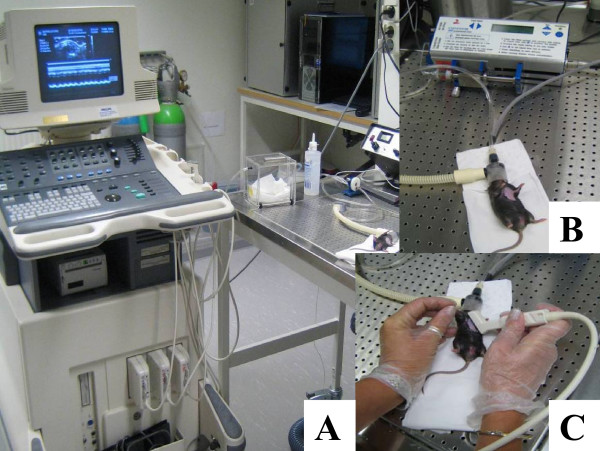
**A-C. Experimental setup**. A. The echocardiographic system. B. Anesthetic setup. C. The high-frequency 15-MHz linear transducer.

The thorax was shaved mechanically and chemically and the mice were placed in a slight left decubitus position on an electrical heating pad (38.5–39°C) to maintain normothermia. All animals were weighed and lightly anesthetized with 1.1–1.2% isoflurane (Abbot Scandinavia AB, Solna) via a nose cone during the echocardiographic examination (Figure 1B).

### Echocardiography

Echocardiography was performed at rest and during dobutamine stress before induction of MI. Examinations was performed using a high-frequency 15-MHz linear transducer (CL 15-7, Philip Medical System, Best) connected to a HDI 5000 ultrasound system (ATL, Philip Medical System, Best) see figure 1A and 1C. All data were stored and later evaluated off-line on an Echo-Pac (Vingmed, Horten) system by one investigator blinded to the animals' identity. For optimal orientation, the long-axis view was first performed, followed by a short-axis view of the pulmonary artery (PA) for pulsed-wave Doppler [10]. Cardiac output (CO) was calculated as CO = PA velocity time integral*π*(0.01/2)^2 ^*HR. From an optimal parasternal short-axis view, two-dimensional targeted M-mode recordings were obtained at the level of the papillary muscles. End-diastolic (LVEDd), end-systolic (LVESd) LV dimensions and posterior wall thickness (LVPWd) were measured according to the leading-edge method of the American Society of Echocardiography [11]. Fractional shortening (FS, %) was calculated as FS = (LVEDd-LVESd)/LVEDd*100. Two-dimensionally guided pulsed Doppler recordings of LV outflow were obtained from the apical "four-chamber" view for measurements of heart rate and ejection time (ET, ms) [12]. Velocity of the circumferential fiber shortening (Vcf c, circ/s) was calculated as Vcf c = ((LVEDd-LVESd)/LVEDd)/ETc(s), where ETc is ET corrected for heart rate. All measurements were based on the average of at least three cardiac cycles.

### Dobutamine stress

Dobutamine (Dobutrex, Eli Lilly Sweden AB, 250 mg) 1 μg/g body weight was given intraperitoneally. Cardiac reserve was investigated 8 min after dobutamine injection.

### Induction of myocardial infarction

**Figure 2:**
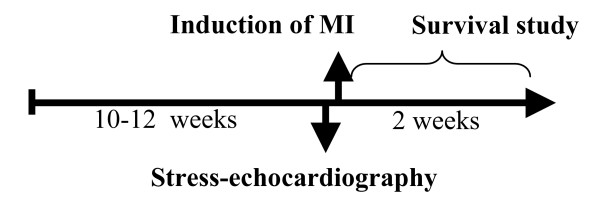
**Study design**. Stress-echocardiography was performed on 10–12-week-old mice. One day after the investigation, myocardial infarction (MI) was induced by ligation of LAD. The animals were then followed for 2 weeks.

To keep the mice sedated and support breathing during the operation, the mice were anesthetized with isoflurane, orally intubated and connected to a respirator (SAR-830 small animal ventilator, GENEQ inc., Montreal, Canada) distributing a mixture of oxygen, air and 2–3% isoflurane. Electrodes were placed on the extremities and connected to an ECG-monitor in order to observe the cardiac rhythm during surgery. An incision was made between the 4^th ^and 5^th ^rib, revealing the upper part of the anterior LV wall and the lower part of the left atrium. An extensive MI was induced by ligation of the left anterior descending (LAD) coronary artery immediately after the bifurcation of the left coronary artery. The efficacy of the procedure was immediately verified by characteristic ECG-changes, and akinesis of the left ventricular anterior wall. After verification of MI the lungs were hyperinflated, positive end-expiratory pressure was applied and the chest was closed. The mice received an i.p. injection of 0.1 ml Temgesic to relieve postoperative pain and the mice recovered spontaneously when the isoflurane was turned off. For study design see figure 2.

### Statistics

Student's t-test was used for statistical analysis of differences between the survivors and the deceased animals. ANOVA was used to evaluate differences among the three groups. Results are expressed as mean ± SD in tables and mean ± SEM in figures. Values of p < 0.05 were considered to be statistically significant. Data were analyzed using SPSS 11.0 for Windows (Chicago, Ill, USA)

## Results

Two weeks after induction of MI, 7 of the 27 mice were alive (26%). The only statistically significant difference in resting values between survivors and deceased animals was a slower heart rate in the surviving animals (R-R interval 183 vs. 138 ms p = 0.02), table 1.

**Table 1:** Left ventricle posterior wall dimension (LVPWd), left ventricle end-diastolic diameter (LVEDd), left ventricle end-systolic diameter (LVESd), fraction shortening (FS), cardiac output (CO), R-R interval (R-R), survivors (S) and deceased (D). Data shown as mean ± SD

Variables at rest	All (n = 27)	Survivors (n = 7)	Deceased (n = 20)	p-value S vs D
LPWd (mm)	0.56 ± 0.07	0.57 ± 0.09	0.56 ± 0.06	0.61
LVEDd (mm)	4.22 ± 0.32	4.25 ± 0.43	4.21 ± 0.28	0.74
LVESDd (mm)	2.73 ± 0.56	2.97 ± 0.59	2.65 ± 0.54	0.19
FS (%)	35.7 ± 9.9	30.7 ± 8.7	37.4 ± 9.9	0.13
CO (ml/min)	14.9 ± 3.3	12.9 ± 3.6	15.6 ± 3.0	0.07
R-R (ms)	150 ± 44	183 ± 70	138 ± 24	0.02

**Figure 3:**
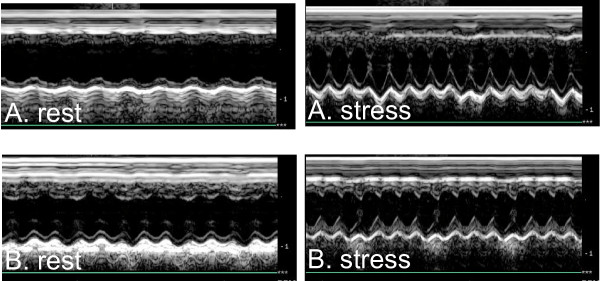
**Native cardiac reserve in WT mice before induction of myocardial infarction**. A. Example of a surviving mouse. FS rest and stress. B. Example of a deceased mouse. FS rest and stress.

**Figure 4:**
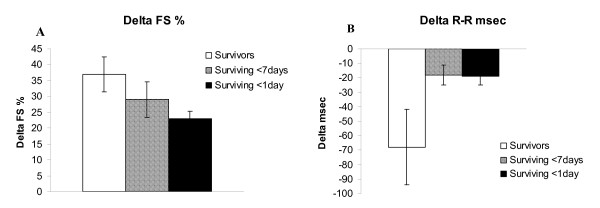
**Native cardiac and chronotropic reserve in WT mice divided according to surviving capacity**. A. Cardiac reserve expressed as delta FS %. ANOVA p < 0.05. B. Chronotropic reserve expressed as delta R-R interval in ms. ANOVA p < 0.05. Data shown as mean ± SEM.

Evaluation of cardiac reserve (stress-rest) revealed that the survivors had increased systolic performance (ΔLVESd p = 0.02), increased cardiac reserve (ΔFS, ΔCO p = 0.02 respectively) and increased chronotropic reserve (ΔR-R interval p < 0.01) during dobutamine challenge in comparison with non-survivors (Figure 3 and table 2). A positive relationship was found between ability to survive and both cardiac (p < 0.05) and chronotropic reserve (p < 0.05) when the mice were divided into three groups: survivors, surviving < 7 days, and surviving < 1 day, (figure 4).

**Table 2:** Left ventricle end-diastolic diameter (LVEDd), left ventricle end-systolic diameter (LVESd), fraction shortening (FS), cardiac output (CO), R-R interval (R-R), survivors (S) and deceased (D). Data shown as mean ± SD

Variables	All (n = 27)	Survivors (n = 7)	Deceased (n = 20)	p-value S vs D
ΔLVEDd (mm)	-0.62 ± 0.32	-0.77 ± 0.38	-0.57 ± 0.29	0.15
ΔLVESd (mm)	-1.43 ± 0.64	-1.86 ± 0.76	-1.28 ± 0.53	0.04
ΔFS (%)	28.2 ± 12.5	37.4 ± 14.4	25.0 ± 10.3	0.02
ΔCO (ml/min)	-0.01 ± 0.38	0.27 ± 0.48	-0.10 ± 0.29	0.02
ΔR-R (ms)	-32 ± 43	-68 ± 68	-19 ± 21	< 0.01

## Discussion

The main finding of the present study is the observation that native inotropic and chronotropic reserve in mice may predict survival during acute MI. To our knowledge, this is the first study to demonstrate a direct correlation between cardiac reserve in healthy animals and survival after acute MI. The chronotropic reserve in humans has been shown in several studies to be a predictor of mortality and morbidity. A Finnish study [8] in healthy middle-aged men showed that survivors have lower resting heart rate but a higher maximal heart rate than individuals who died of cardiovascular mortality during a follow up of 11 years. Similarly, Cheng *et al *[7] has shown in healthy men (27459) that chronotropic reserve is inversely associated with cardiovascular mortality. In particular, low chronotropic reserve in healthy younger men seems to be associated with future cardiovascular events. However, cardiac reserve as described in this study was not previously evaluated in healthy individuals for prediction of mortality. DSE has been shown to be an independent prognostic predictor of all-cause mortality and hard cardiac events in elderly patients. On the other hand, 47% of these patients showed signs of ischemia during the DSE and therefore this population can not be defined as a normal healthy population [5].

To determine whether native cardiac and chronotropic reserve can predict survival after MI, we used a previously well-defined mouse model of acute MI [13] and DSE [14]. Each animal has been investigated with DSE prior to induction of MI. The analysis of the native inotropic and chronotropic reserve showed clear differences between the deceased mice and survivors. No differences were found between the groups at rest except for a lower R-R interval and a trend towards a lower CO in the survivors. Although the surviving animals seemed to function at somewhat lower resting capacity, they showed improved ability to increase heart rate and mechanical function during stress, demonstrating a clear association between survival and inotropic and chronotropic reserve. Even if it may seem logical that only the "fittest" animals survive a large MI, this study does not provide the explanation for the observed results and we can only speculate about the mechanisms.

While humans are characterised by general variability in the coronary artery anatomy, inbred mice (C57BL6) have a relatively uniform coronary tree anatomy [13]. Ligation of the LAD immediately after the bifurcation results in an extensive infarction of the free wall extending down to the apex, with sparing of the septum. Salto-Tellez *et al *could also show that ligation of the LAD ~1 mm after the bifurcation results in a consistent size of the infarction. Although, the infarct size of the deceased animals in this study is unknown, all survivors had large MI with comparable reduction in systolic function (data not shown). Using the same ligation technique, mice in our experience develop approximately the same mean infarct size (unpublished data).

We believe the explanation for our results showing that the surviving animals have a better native cardiac function may be found in the heterogeneity of the normal myocardium. It has been shown previously that even genetically homogenous groups of animals with seemingly uniform macroscopic characteristics of the heart show substantial physiological heterogeneity of protein expression, energy turnover and flow [15]. It would be of interest to evaluate the possible prenatal influence on native myocardial function and heterogeneity. Some animal studies have already addressed this issue in regard to other physiological and pathophysiological variables [16,17]. However, another mechanism may explain the difference in the survival rate in respect to infarct size. It is possible that the presence of higher cardiac reserve indicates the ability of the myocardium to develop smaller infarctions upon ischemic injury, which results in lower acute mortality. It is also possible that a reduction in the β-1 adrenoceptor density or sensitivity could account for a reduced inotropic and/or chronotrophic reserve.

Regardless of the mechanisms involved, the predictive value of cardiac reserve is not diminished. An important aspect of our results is that the association between cardiac reserve and post-MI mortality was observed in healthy animals. In previous human studies, it could be anticipated that the existence of reduced cardiac reserve would be a sign of subclinical cardiac disease. Because different mechanisms may be involved in the improved survival in the presence of higher cardiac reserve, further studies are clearly needed to clarify this novel observation.

We conclude that the surviving animals have a better native cardiac function before MI induction compared with the deceased animals. This indicates that native cardiac and chronotropic reserve may be important determinants and predictors of survival in the setting of large MI and early post-infarction heart failure. Future studies should address the mechanisms behind these findings.

## Competing interests

The author(s) declare that they have no competing interests.

## Authors' contributions

MST carried out the echocardiographic examinations and evaluations, performed the statistical analysis and drafted the manuscript. TR and ML carried out the myocardial infarctions and have also participated in the echocardiographic examinations, evaluations, statistical analysis and in drafting the manuscript. EO has provided the scientific rationale (together with MST), was responsible for practical, scientific conduction of the study and revised the final draft of the manuscript. All authors have read and approved the final manuscript.
